# Differing prevalence of microcephaly and macrocephaly in male and female fetuses

**DOI:** 10.3389/fgwh.2023.1080175

**Published:** 2023-02-24

**Authors:** Amalia M. Brawley, Eric W. Schaefer, Elizabeth Lucarelli, Serdar H. Ural, Cynthia H. Chuang, Wenke Hwang, Ian M. Paul, Carrie Daymont

**Affiliations:** ^1^ Department of Obstetrics and Gynecology, Penn State College of Medicine, Hershey, PA, United States; ^2^ Department of Public Health Sciences, Penn State College of Medicine, Hershey, PA, United States; ^3^ Department of Medicine, Penn State College of Medicine, Hershey, PA, United States; ^4^ Department of Pediatrics, Penn State College of Medicine, Hershey, PA, United States

**Keywords:** microcephaly, macrocephaly, ultrasound, biometry, growth

## Abstract

**Objective:**

To compare the proportion of female and male fetuses classified as microcephalic (head circumference [HC] < 3rd percentile) and macrocephalic (>97th percentile) by commonly used sex-neutral growth curves.

**Methods:**

For fetuses evaluated at a single center, we retrospectively determined the percentile of the first fetal HC measurement between 16 and 0/7 and 21–6/7 weeks using the Hadlock, Intergrowth-21st, and NICHD growth curves. The association between sex and the likelihood of being classified as microcephalic or macrocephalic was evaluated with logistic regression.

**Results:**

Female fetuses (*n* = 3,006) were more likely than male fetuses (*n* = 3,186) to be classified as microcephalic using the Hadlock (0.4% male, 1.4% female; odds ratio female vs. male 3.7, 95% CI [1.9, 7.0], *p* < 0.001), Intergrowth-21st (0.5% male, 1.6% female; odds ratio female vs. male 3.4, 95% CI [1.9, 6.1], *p* < 0.001), and NICHD (0.3% male, 1.6% female; odds ratio female vs. male 5.6, 95% CI [2.7, 11.5], *p* < 0.001) curves. Male fetuses were more likely than female fetuses to be classified as macrocephalic using the Intergrowth-21st (6.0% male, 1.5% female; odds ratio male vs. female 4.3, 95% CI [3.1, 6.0], *p* < 0.001) and NICHD (4.7% male, 1.0% female; odds ratio male vs. female 5.1, 95% CI [3.4, 7.6], *p* < 0.001) curves. Very low proportions of fetuses were classified as macrocephalic using the Hadlock curves (0.2% male, < 0.1% female; odds ratio male vs. female 6.6, 95% CI [0.8, 52.6]).

**Conclusion:**

Female fetuses were more likely to be classified as microcephalic, and male fetuses were more likely to be classified as macrocephalic. Sex-specific fetal head circumference growth curves could improve interpretation of fetal head circumference measurements, potentially decreasing over- and under-diagnosis of microcephaly and macrocephaly based on sex, therefore improving guidance for clinical decisions. Additionally, the overall prevalence of atypical head size varied using three growth curves, with the NICHD and Intergrowth-21st curves fitting our population better than the Hadlock curves. The choice of fetal head circumference growth curves may substantially impact clinical care.

## Introduction

Fetal biometry is an important component in understanding overall fetal well-being. Deviations from typical growth may prompt further evaluation, possibly affecting delivery planning, and ultimately, neonatal outcomes (1). Growth curves are used to determine if biometric measurements are considered typical for a given gestational age; the data used to create growth curves and the population in which these curves are used can impact how these measurements are interpreted. Starting at birth, infant size measurements are evaluated using sex-specific growth curves, reflecting small but consistent growth differences between sexes (2). However, fetal growth curves are not typically interpreted in a sex-specific manner.

Our study focuses on one component of biometry, head circumference (HC), and the potential implications of using sex-neutral growth curves to interpret fetal HC. There are no universally accepted definitions for microcephaly and macrocephaly; however, < 3rd and > 97th percentiles are commonly accepted as the cutoffs for microcephaly and macrocephaly, respectively. The mean HC for male and female fetuses differs by 0.3–0.5 standard deviations (SDs), with males having larger heads, on average, than females. However, most fetal growth curves are not sex-specific. These differences in growth can impact how many fetuses of each sex are classified as having microcephaly or macrocephaly, possibly resulting in over- or under-diagnosis depending on sex.

If fetal HC follows a normal distribution, the difference in means between male and female HC allows us to predict the difference in the proportions of male and female fetuses with microcephaly and macrocephaly. However, not all fetal and infant growth curves follow a normal distribution for HC (3, 4). For example, two populations with the same mean and standard deviation, one distributed normally and one left-skewed, would have different values for <3rd and >97th percentiles. Therefore, knowing the difference in mean HC between male and female fetuses does not necessarily allow us to predict precisely how the proportions of male and female fetuses at the extremes of a distribution differ.

As illustrated during the Zika virus (ZIKV) epidemic, whether or not a fetus is classified as microcephalic can significantly impact evaluation and management (5). ZIKV is a single-stranded RNA virus that is often asymptomatic (6); when symptomatic, it generally causes a mild illness (7). ZIKV infection during pregnancy has been shown to cause nervous system abnormalities, including microcephaly, in some fetuses (8). The ZIKV epidemic highlights the importance of accurate evaluation of fetal HC. As described above, prenatal growth curves are not sex-specific, despite the fact that size differences between male and female fetuses have been shown to be clinically significant (9–12). Current diagnostic criteria for fetal HC are limited by the widespread use of sex-neutral growth curves. In this paper, we applied three commonly used sex-neutral growth curves to our population: Hadlock, Intergrowth-21st, and NICHD.

The Hadlock curves were published in 1984 as a reference based on a study of 361 pregnant women in Houston and continue to be widely used (13). They allow determination of HC *z*-scores using a cubic equation for mean HC by age and a non-varying standard deviation (SD). The 2014 Intergrowth-21st standards are based on data from eight different countries and allow determination of HC z-scores based on fractional polynomials for median HC and SD as a function of GA (14). Also released recently, the 2015 NICHD growth standards were based on data prospectively collected from 12 sites across the United States. NICHD published percentiles using quantile regression for each week of GA and separate growth curves for four race/ethnicity groups (3).

Evaluating real-world differences in the proportions of male and female fetuses classified as having atypical head size is an important step in understanding the clinical implications of using sex-neutral vs. sex-specific fetal growth curves and whether it is important to incorporate sex-specific curves when interpreting fetal growth. If proportions of males and females classified as microcephalic and macrocephalic differ from what we expect based on the means, this may also provide some information about the epidemiology of causes of microcephaly and macrocephaly in a population.

We hypothesize that more female than male fetuses meet criteria for microcephaly and more male than female fetuses meet criteria for macrocephaly when classifying second trimester HC measurements using three sex-neutral growth curves.

## Materials and methods

We performed a retrospective cohort study of pregnant women seen at Penn State Milton S. Hershey Medical Center (HMC) who delivered between 7/1/2012 and 6/30/2017. The Penn State Institutional Review Board approved the study and provided a waiver of informed consent (study number 00007195).

### Data extraction

We obtained prenatal ultrasound data for all women who gave birth at HMC in the specified time frame and had at least one ultrasound at HMC during their pregnancy. Fetal measurements and estimated gestational age (GA) were extracted from a dedicated database. Estimated GA was determined by clinicians based on American College of Obstetricians and Gynecologists (ACOG) guidelines (15). We included ultrasounds of singleton pregnancies only and excluded ultrasounds without data for GA or number of fetuses. For each pregnancy, we selected the index ultrasound, which was the first ultrasound that occurred between 16 and 0/7 and 21–6/7 weeks gestation with a non-missing value for HC. Ultrasound measurements at earlier and later GAs were recorded sporadically and therefore not included in our analysis. We excluded pregnancies without an index ultrasound from further analysis. We extracted data from the electronic health record (EHR) to determine maternal race and ethnicity.

To determine the sex of the fetuses, we linked infants to mothers using a birth log containing data manually recorded by the Labor and Delivery nurses at each delivery. These data include maternal and infant identifiers and infant sex. Data from the EHR were used to confirm the infant sex found on the birth log. The sex from the EHR was used when these were discordant.

### Data linking

**Figure 1:**
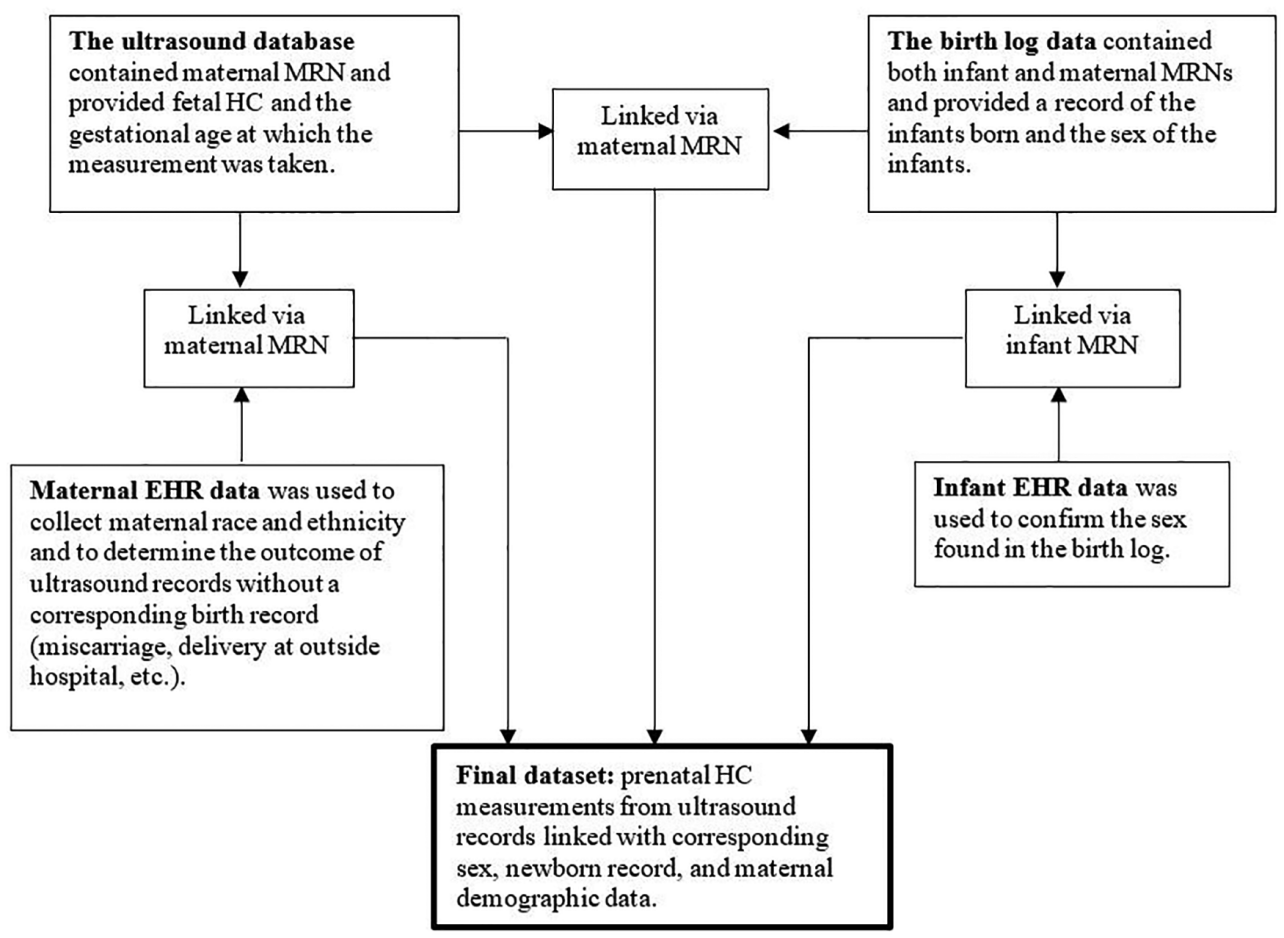
Four separate datasets. Schematic representation of methods providing an overview of how four separate datasets were used to create a final dataset. MRN = medical record number; HC = head circumference; EHR = electronic health record.

Ultrasound visits were linked with delivery data from the birth log using maternal medical record number (MRN), expected date of delivery, and actual delivery date (Figure 1). We linked ultrasounds to a birth if the actual delivery date was within ±21 days of the expected date of delivery corrected for estimated GA at birth as recorded in the birth log. We included term and preterm births; a 21-day window was used to ensure the ultrasound data were linked with the correct baby. Some birth records (*n* = 448) had a missing GA at delivery. For these records, the GA at admission was used; in records for which GA was available at admission and delivery, the difference was larger than three days in only 2.9% of admissions. The resulting dataset included one or more ultrasound visits linked to one pregnancy and one delivery record in the birth log; for this analysis, we included only the index ultrasound as described above.

In the time frame specified above, the ultrasound database contained 52,691 unique ultrasounds. Of these, 30,870 were linked to the birth log *via* maternal MRN, leaving 21,821 unlinked ultrasounds. To confirm that our linking methods were appropriate, we reviewed charts of 400 unlinked pregnancies with an estimated delivery date within the date range for which birth logs were available. Chart review data was stored in REDCap (16). The majority (89%) of unlinked pregnancies ended in miscarriage/termination or birth at another hospital; only 11% appeared to be due to missing data or errors (e.g., mistyping a medical record number) in the birth log. Even with chart review, we could not determine with certainty whether these pregnancies were linked to a given birth, so we excluded all unlinked pregnancies. We also excluded ultrasounds from pregnancies that were linked with a birth log record for which sex was unavailable.

### Outcome definitions

To determine whether classification differences were consistent across growth curves, we classified HC using three different growth curves. We used growth curves that are either commonly used (Hadlock and Intergrowth-21st) and/or were designed to be representative of the U.S. population (Intergrowth-21st and National Institute of Child Health and Human Development [NICHD]) to determine the proportion of female and male fetuses classified as having microcephaly (<3rd percentile, z-score < −1.88) or macrocephaly (>97th percentile, z-score >1.88) in the data subset of our sample described above. While the Society for Maternal-Fetal Medicine (SMFM) provides recommendations for standardizing the evaluation of fetal HC in the context of Zika virus exposure (5), there are no universal definitions for microcephaly and macrocephaly; the 3rd and 97th percentiles were chosen because they are commonly used and because the information provided in the NICHD curves does not allow direct calculation of other potential cutpoints for microcephaly and macrocephaly. All three evaluated growth curves are sex-neutral, using a single set of curves for both sexes.

We used cubic interpolation to calculate values of the 3rd and 97th percentiles for integer values of GA in days (17). NICHD percentiles were published separately for four specific race/ethnicity groups: Asian/Pacific Islander, Hispanic, Black non-Hispanic, and White non-Hispanic. There were no published NICHD percentiles that were nonspecific for race/ethnicity. We used the mean of the four race/ethnicity-specific values at each GA to create percentiles for a fifth group, deemed “Uncategorized.” For the NICHD analyses only, we excluded the small number of ultrasounds that could not be linked with maternal data. Women with a recorded race/ethnicity that did not fit in these categories, or whose maternal data were available but missing race/ethnicity data, were evaluated using the “Uncategorized” percentiles. Although there are limits of the reliability and precision of race/ethnicity data, the EHR was the only potential source of race/ethnicity data for this population and therefore the only way to evaluate our data compared to the US-based NICHD percentiles.

### Inclusion and exclusion criteria

We did not exclude pregnant women with comorbidities or demographic factors that can be associated with having fetuses at either of the growth extremes. It was important to include these women to evaluate the real-world sex differences in atypical head size. Additionally, we did not have sufficient data to identify all of these conditions or factors. Specifically, we did not perform a sensitivity analysis excluding women with obesity from our analysis because pre-pregnancy body mass index (BMI) was often not available in the EMR; therefore, we could not reliably differentiate women with obesity from women without obesity.

We did not include postnatal outcomes in this analysis, as we could not accurately identify these outcomes. Many pathologic causes of atypical head size are not identified during the birth hospitalization (18, 19), an issue that may be more prominent among those with a HC at the edge of the typical range that were the focus of our study. We studied patients at a tertiary referral center with a wide catchment area and would have missed a substantial proportion of infants who had diagnoses made after discharge. Prenatal concerns can also influence postnatal care. Therefore, increased frequency of prenatal microcephaly in females and prenatal macrocephaly in males could bias the proportion of infants identified with pathologic causes of atypical head size. There are no accepted lists of conditions that cause atypical head size or guidelines regarding identification of such conditions. Finally, preliminary work in our institution revealed significant differences among clinicians regarding the likelihood that certain conditions caused atypical head size starting in the second trimester.

### Statistical analysis

Logistic regression was used to evaluate the association between fetal sex and the likelihood of having microcephaly and macrocephaly, evaluated separately. To account for women with more than one pregnancy within the study time frame, we used a mixed effects logistic regression model for each outcome. A fixed effect for fetal sex and a random effect for mother were included in these models.

The software program used for statistical analysis was R 3.5.1. In a power analysis conducted before the data were extracted, a sample size of 10,000 pregnancies yielded >99% power to detect a difference under the relatively conservative estimate that female fetuses had twice the prevalence of microcephaly as male fetuses given a difference of 0.3 SD between male and female HC means.

## Results

**Figure 2:**
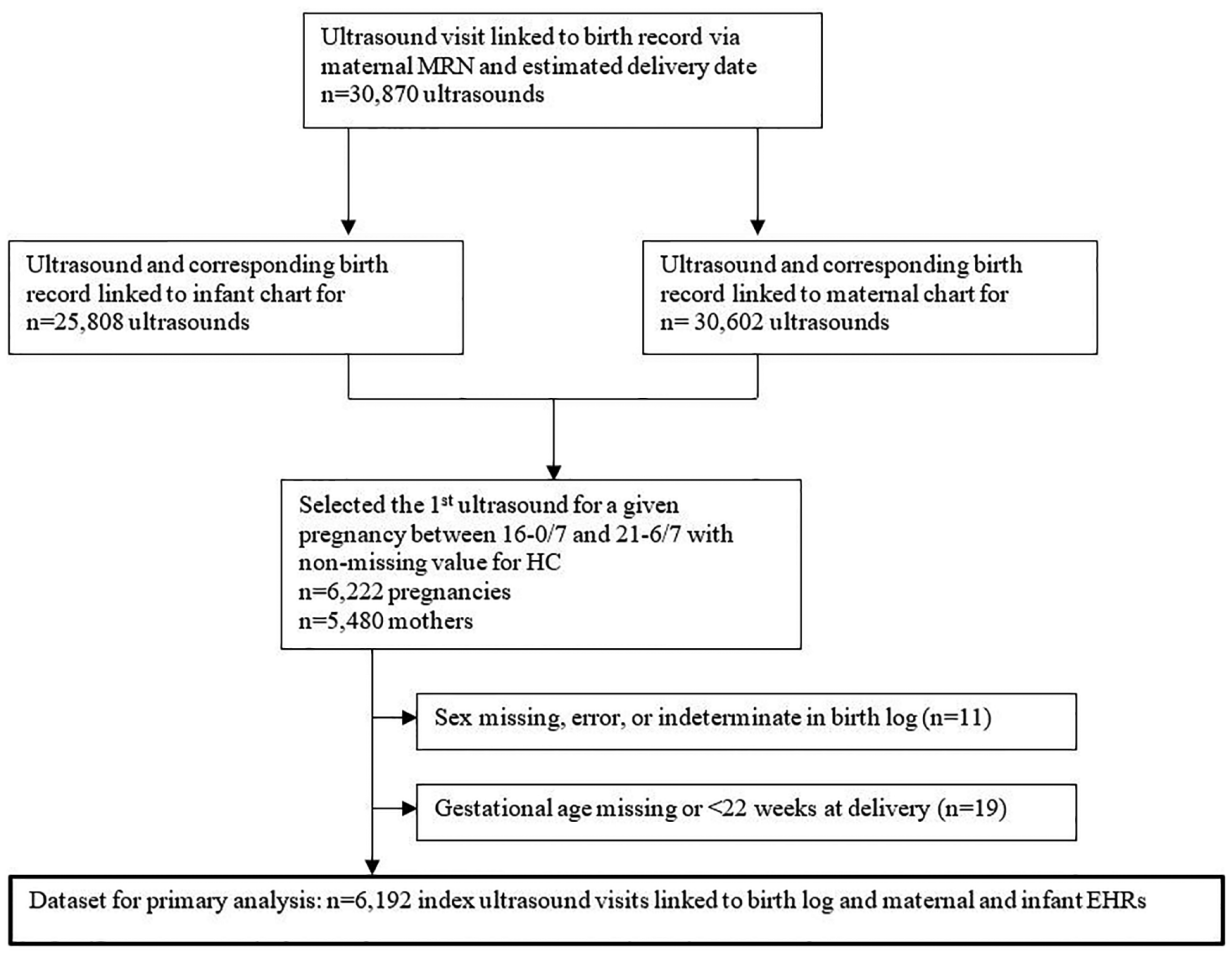
Final dataset for analysis. Schematic representation of how the final dataset for analysis was derived. MRN = medical record number; HC = head circumference; EHR = electronic health record.

The final dataset for analysis contained 6,192 unique ultrasound visits for 5,454 women (Figure 2). Four percent of ultrasound visits occurred between 16 and 0/7 and 17–6/7 weeks gestation, 63.8% between 18 and 0/7 and 19–6/7 weeks gestation, 26.6% between 20 and 0/7 and 20–6/7 weeks gestation, and 5.6% between 21 and 0/7 and 21–6/7 weeks gestation. The sex recorded in the birth log indicated 3,186 male fetuses (51.5%) and 3,006 female fetuses (48.5%). For the 5,013 records that were linked to infant EHR data *via* newborn MRN, the EHR and birth log listed the same sex in 99.3% of the records. For the 6,156 records (99.4%) that were linked to the maternal EHR data, Table 1 shows the final maternal race/ethnicity groups used for the NICHD growth curve. Very few recorded HC values appeared implausible for GA; all HC values were retained in the analysis.

**Table 1:** Maternal race/ethnicity.

	Total (*N* = 6156)
**Maternal race**	
American Indian/Alaska Native	8 (0.1%)
Asian	155 (2.5%)
Black or African American	434 (7.1%)
Native Hawaiian or Pacific Islander	4 (0.1%)
White (Caucasian)	4,914 (79.8%)
Other Race	525 (8.5%)
Patient Declined	1 (<0.1%)
Two or More Races	113 (1.8%)
Unavailable	2 (<0.1%)
**Maternal ethnicity**	
Hispanic, Latino, or Spanish Origin	443 (7.2%)
Not Hispanic, Latino, or Spanish Origin	5,698 (92.6%)
Patient Declined	2 (<0.1%)
Unavailable	13 (0.2%)
**Maternal race/ethnicity for NICHD reference chart**	
Asian	155 (2.5%)
Hispanic	440 (7.1%)
Black, non-Hispanic	423 (6.9%)
White, non-Hispanic	4,839 (78.6%)
Other (including missing)	299 (4.9%)

Female fetuses were more likely than male fetuses to be classified as microcephalic using the Hadlock (0.4% male, 1.4% female; OR F vs. M 3.7, 95% CI [1.9, 7.0], *p* < 0.001), Intergrowth-21st (0.5% male, 1.6% female; OR F vs. M 3.4, 95% CI [1.9, 6.1], *p* < 0.001), and NICHD (0.3% male, 1.6% female; OR F vs. M 5.6, 95% CI [2.7, 11.5], *p* < 0.001) curves (Table 2). Male fetuses were more likely than female fetuses to be classified as macrocephalic using the Intergrowth-21st (6.0% male, 1.5% female; OR M vs. F 4.3, 95% CI [3.1, 6.0], *p* < 0.001) and NICHD (4.7% male, 1.0% female; OR M vs. F 5.1, 95% CI [3.4, 7.6], *p* < 0.001) curves (Table 2). The overall prevalence of macrocephaly using the Hadlock curve was low, and the difference in prevalence by sex was not statistically significant.

**Table 2:** Prevalence of microcephaly and macrocephaly by sex.

Growth Curve	Female Fetuses (*N* = 3006)	Male Fetuses (*N* = 3186)	Odds Ratio and 95% CI
	**<3rd percentile**	**<3rd percentile**	**Female vs. Male**
Hadlock	41 (1.4%)	12 (0.4%)	3.7 [1.9, 7.0]
Intergrowth-21st	48 (1.6%)	15 (0.5%)	3.4 [1.9, 6.1]
NICHD*	47 (1.6%)	9 (0.3%)	5.6 [2.7, 11.5]
** **	**>97th Percentile**	**>97th Percentile**	**Male vs. Female**
Hadlock	1 (<0.1%)	7 (0.2%)	6.6 [0.8, 52.6]
Intergrowth-21st	44 (1.5%)	192 (6.0%)	4.3 [3.1, 6.0]
NICHD*	29 (1.0%)	150 (4.7%)	5.1 [3.4, 7.6]

* NICHD included 6,157 total fetuses (2,991 female and 3,166 male fetuses).

**Figure 3:**
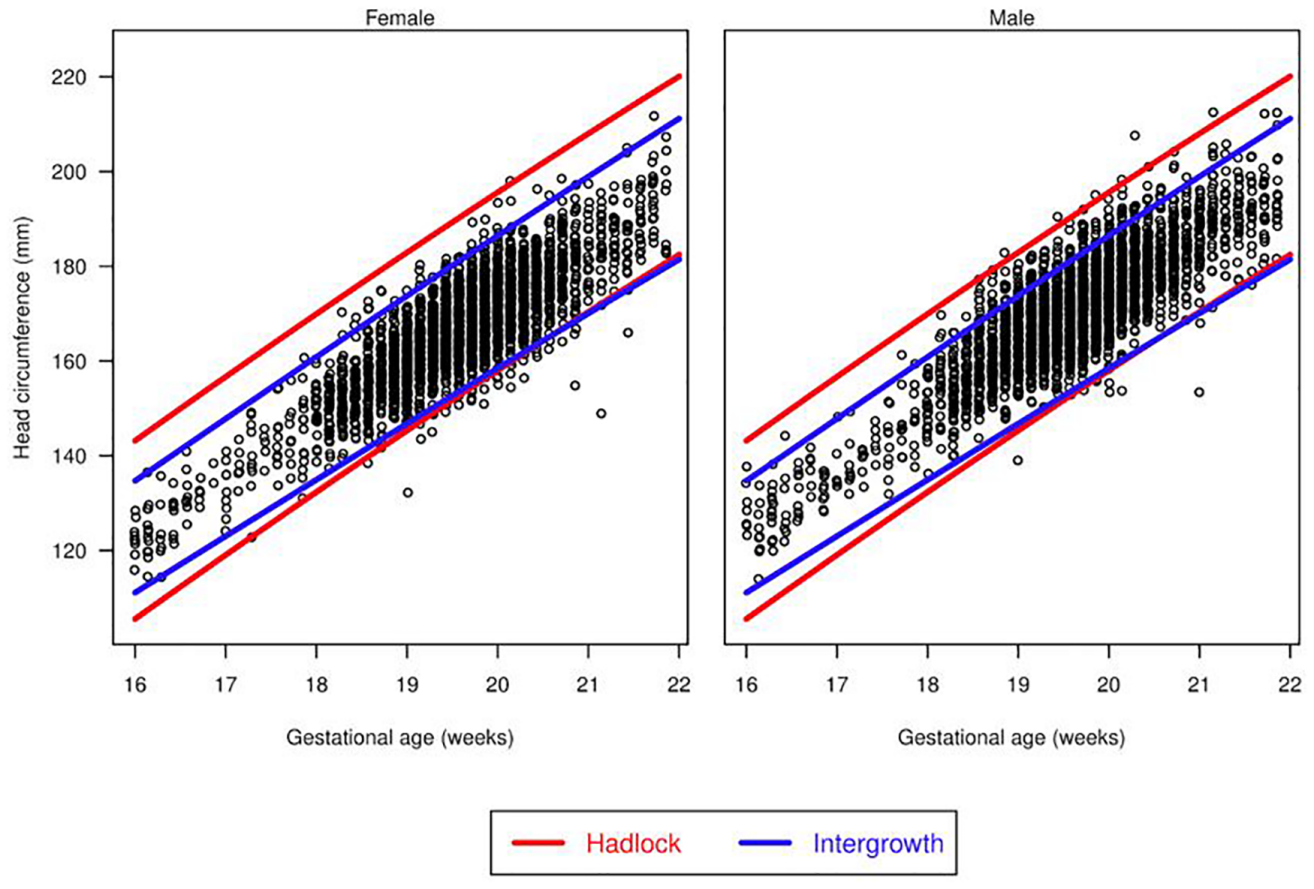
Head circumference by gestational age. Scatter plots, separated for males and females, of fetal head circumference by gestational age at time of ultrasound with percentiles (3rd and 97th) overlaid for Hadlock and Intergrowth-21st charts.

**Figure 4:**
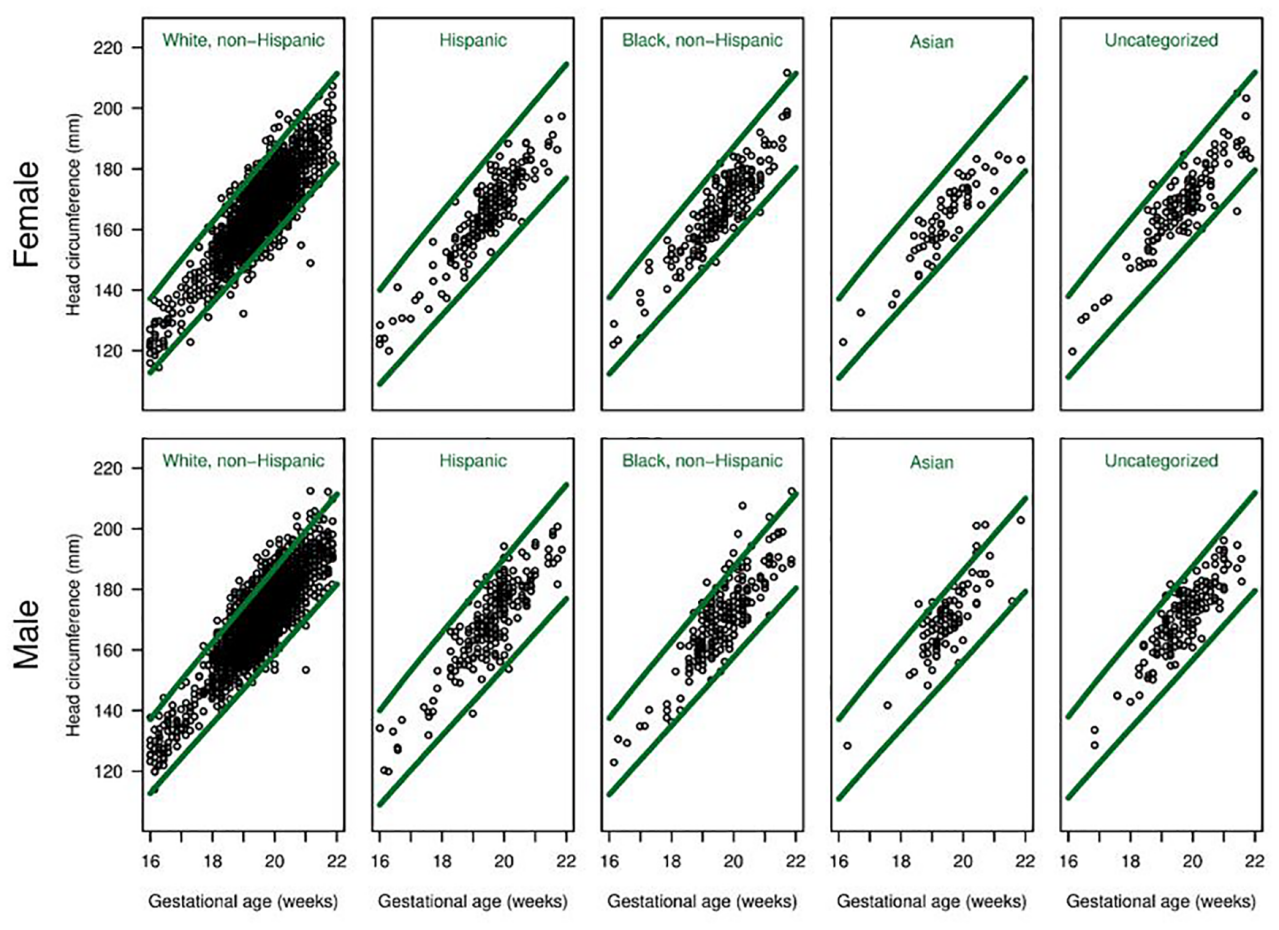
Head circumference by gestational age for each race/ethnicity group. Scatter plots, separated for males and females, of fetal head circumference by gestational age at time of ultrasound for each race/ethnicity category used in NICHD charts. Percentiles (3rd and 97th) are overlaid (green lines) for NICHD reference charts.

There were differences in the overall proportions of fetuses classified as having microcephaly or macrocephaly depending on the growth curve used (Figure 3, Figure 4 and Table 3). Figure 3 shows scatter plots, separated by males and females, of fetal HC by gestational age with percentiles (3rd and 97th) overlaid for Hadlock and Intergrowth-21st growth charts. Figure 4 shows scatter plots, separated by male, female, and race/ethnicity, of fetal HC by gestational age percentiles (3rd and 97th) for each race/ethnicity category used in NICHD growth charts. These visuals help highlight the difference in the number of females vs. males that are classified as microcephalic or macrocephalic by the Hadlock, Intergrowth-21st, and NICHD growth curves.

**Table 3:** Overall prevalence of microcephaly and macrocephaly.

Growth Curve	Total Fetuses (*N* = 6192)
	**<3rd percentile**
Hadlock	53 (0.9%)
Intergrowth-21st	63 (1.0%)
NICHD*	56 (0.9%)
** **	**>97th Percentile**
Hadlock	8 (0.1%)
Intergrowth-21st	236 (3.8%)
NICHD	179 (2.9%)

* NICHD included 6,157 total fetuses (2,991 female and 3,166 male fetuses).

## Discussion

Consistent with our hypotheses, we found that second-trimester female fetuses were approximately three times more likely than male fetuses to be classified as having microcephaly, and second-trimester male fetuses were approximately three times more likely than female fetuses to be classified as having macrocephaly when applying sex-neutral growth curves to our population. We also found wide variation in the proportion of fetuses meeting criteria for microcephaly or macrocephaly based on second-trimester measurements depending on which sex-neutral growth curve was applied to our population: Hadlock, Intergrowth-21st, or NICHD.

Prior studies have demonstrated differences in growth curves created separately for male and female fetuses (20, 21), and studies of various fetal growth parameters have demonstrated the clinical significance of sex differences (9–12). Sex-specific estimated fetal weight (EFW) curves predict stillbirth better than sex-neutral curves, and the between-sex differences for EFW are smaller than those for HC (approximately 0.2 SDs at 20 weeks) (9).

Rather than predicting expected proportions based on the mean and SD of a normal distribution, our study evaluated how many fetuses actually met criteria for microcephaly and macrocephaly when three different sex-neutral growth curves were applied to our population. This is important because HC does not necessarily follow a normal distribution and does not necessarily meet other criteria (identical SDs) required for predicting proportions from differences in means.

Our analysis, driven partially by the need to better understand prenatal diagnosis of microcephaly in the context of the recent Zika virus epidemic, demonstrates that ignoring known differences in fetal growth by sex can result in large differences in the proportion of male and female fetuses classified as having microcephaly or macrocephaly. We are unaware of any evidence of a true difference by sex in the prevalence of pathology that causes atypically small or large heads. Therefore, any discrepancy that we see in the proportions of male and female fetuses classified with microcephaly and macrocephaly is likely due to the methods used to interpret fetal growth. This artifact could lead to significant potential consequences.

There were significant differences by sex in the proportions of fetuses in our population classified as macrocephalic and microcephalic. When a fetus with a pathological condition associated with abnormal head size has a falsely normal HC measurement, the opportunity for early detection that could support further prenatal and/or neonatal planning may be missed. Alternatively, a healthy fetus with a somewhat large or small head may be subjected to unnecessary testing, which is not only burdensome to the patient, but also associated with significant maternal anxiety (22–24).

In addition to differences by sex, there were differences in the proportions of fetuses classified as microcephalic or macrocephalic depending on which growth curve was used. When all fetuses are evaluated together, the proportion of fetuses meeting criteria for macrocephaly using the NICHD and Intergrowth-21st curves was close to the expected proportion of 3%. Using the Hadlock curve, however, very few fetuses met this criterion. For all three curves, the proportion of fetuses meeting criteria for microcephaly was approximately 1/3 the expected proportion of 3%. This difference in proportions could reflect a real difference in the distribution of fetal HC at our institution compared to the populations underlying the evaluated growth curves or could represent measurement differences at our center for fetuses with smaller heads. If this under-identification of microcephalic fetuses is present at other centers, it is possible that a significant number of fetuses with prenatally identifiable pathology are currently not being identified.

Prior to recent advancements in prenatal sex determination, the challenge of accurately determining fetal sex made the utility of sex-specific fetal growth curves irrelevant for practical purposes. However, it is now technically feasible to use cell-free DNA screening to accurately and safely determine fetal sex (25, 26). Sex-specific growth curves could be useful in the subset of fetuses with a HC that is atypical or borderline atypical on a sex-neutral curve, such as <10th or >90th percentile. In those cases, determining fetal sex could allow evaluation using a sex-specific growth curve, thereby directing further evaluation, counseling, and possible intervention in a more informed context. Further research would be needed to evaluate the risks and benefits of this approach.

The poor fit of the Hadlock curves is important to note as it is one of the most widely used growth curves in clinical practice. The Hadlock curves were developed in 1984 based on data from a relatively small number (361) of women, all Caucasian and from the same area. Our findings support use of a more recent growth curve.

The primary strength of this study is the use of accurate sex data from birth to evaluate differences by sex in a relatively large population. The primary limitations are that it was done retrospectively in a single center and that we lacked data on long-term clinical outcomes.

In conclusion, applying sex-neutral fetal growth curves to second-trimester HC measurements classifies more female fetuses as microcephalic and more male fetuses as macrocephalic. Our findings support use of a growth curve more current than Hadlock for evaluation of HC in fetuses in our population. In the future, the creation of widely applicable sex-specific HC references to be used for fetuses with a HC at the edge of the expected range could improve prenatal identification of, and subsequent intervention or counseling for, fetuses with an atypical head size.

## Data Availability

The data that support the findings of this study are not publicly available due to privacy and ethical restrictions. Upon request, the corresponding author will assist others in contacting the relevant parties to obtain approval to analyze the data.
